# Impact of patent foramen ovale on transvenous lead extraction characteristics and outcomes

**DOI:** 10.1016/j.hroo.2026.02.024

**Published:** 2026-03-06

**Authors:** Aaron Park, Nolan Martin, John Power, Omar Aldaas, Gordon Ho, Timothy Maus, Ulrika Birgersdotter-Green, Travis Pollema

**Affiliations:** 1 University of California, San Diego, La Jolla, California; 2 Mount Sinai Hospital, New York, New York

**Keywords:** Transvenous lead extraction (TLE), Patent foramen ovale (PFO), Cardiac implantable electronic devices (CIEDs), Stroke, Mortality, Transesophageal echocardiogram (TEE), Complications

## Abstract

**Background:**

Transvenous lead extraction (TLE) may be indicated for cardiac implantable electronic device infection, malfunction, or upgrade. TLE can increase right heart pressures and mobilize debris, resulting in a right-to-left embolism and stroke via a patent foramen ovale (PFO).

**Objective:**

This study aimed to identify the prevalence of PFO in patients undergoing TLE and compare the incidence of adverse events.

**Methods:**

Intraoperative transesophageal echocardiogram is routinely performed during TLE at the University of California, San Diego, to identify PFO. Between June 2020 and October 2025, 283 patients underwent 289 lead extraction procedures and had intraoperative transesophageal echocardiogram reports available. Patients were compared by PFO status.

**Results:**

Of 283 patients, the mean age was 64.98 years (standard deviation ± 15.17); 65.74% were male. PFO was detected in 37 patients (12.80%) but was not associated with different baseline characteristics. Overall, the mean lead count was 2.14 ± 0.51, dwell time was 4.91 ± 4.25 years, and procedural success was 90.66% (odds ratio [OR] 0.43; 95% confidence interval 0.16–1.19; *P* = .106). The most common extraction indications were malfunction (45.67%) and infection (30.45%). Primary endpoints were in-hospital stroke (1 of 37 [2.7%] in PFO cohort vs 1 of 252 [0.40%]; OR 3.81; 95% confidence interval 0.18–79.38; *P* = .388), myocardial infarction (none in either cohort), and all-cause mortality (2 of 37 [5.41%] in PFO cohort vs 0 of 252 [0%] in no PFO cohort; Fisher’s exact test *P* = .016; unable to calculate OR with multivariate regression). Of these 2 deaths in the PFO cohort, 1 was caused by stroke-related complications and the other by sepsis.

**Conclusion:**

Stroke incidence was not significantly different by PFO status, but mortality was significantly higher in those with PFO.

**Key Findings**

▪ The overall in-hospital mortality rate during transvenous lead extraction (TLE) was significantly higher in the patent foramen ovale (PFO) cohort (5.41%) than the non-PFO cohort (0%), with a statistically significant difference (*P* = .016); multivariate regression was unable to be performed to calculate an odds ratio given that there were no events in the non-PFO cohort.
▪ The incidence of in-hospital stroke was numerically higher in the PFO cohort (2.70%) than the non-PFO cohort (0.40%), but this difference was not statistically significant (*P* = .388); this remained true using multivariate regression analysis, with an odds ratio of 3.81 (95% confidence interval 0.18–79.38; *P* = .388).
▪ Complete procedural success (90.66% overall) was lower in the PFO cohort (83.78%) than in the non-PFO cohort (91.67%), although this difference was not statistically significant (*P* = .106); this remained true using multivariate regression analysis, with an odds ratio of 0.43 (95% confidence interval 0.16–1.19; *P* = .106).
▪ A PFO was detected in 12.80% of patients (37 of 283) undergoing TLE using routine intraoperative transesophageal echocardiogram.

## Introduction

Transvenous lead extraction (TLE) is a procedure performed in patients with cardiac implantable electronic devices (CIEDs), including pacemakers and defibrillators, for indications including device infection, lead malfunction, system upgrade, and chronic device-related symptoms such as pain or discomfort. Although TLE is a generally safe procedure, it carries inherent risks, including complications such as myocardial infarction, stroke, and procedural mortality. 1 of the most concerning potential complications is embolism, which can occur when thrombus or other debris is dislodged during the procedure and travels to vital organs, particularly the brain. Owing to these risks, understanding the factors that may influence outcomes during TLE is essential for improving patient safety and procedure success.

Patent foramen ovale (PFO) is a congenital cardiac anomaly characterized by a persistent opening between the right and left atria, which typically closes soon after birth. However, in some patients, the PFO remains patent throughout life. The presence of PFO has been associated with an increased risk of paradoxical embolism and stroke, particularly in the setting of other risk factors such as deep vein thrombosis or procedural manipulation. Several studies have suggested that PFO may contribute to worse outcomes in other clinical contexts, such as during cardiac surgery or invasive procedures, where the risk of embolic events may be heightened.^1^^,^^2^ These findings have raised questions about whether PFO might also contribute to complications during TLE, a procedure that involves significant manipulation of the heart and vascular structures, as well as temporary alteration of intracardiac hemodynamics.

However, the specific impact of PFO on procedural outcomes in TLE remains poorly understood. Although previous studies have identified associations between PFO and embolic events in certain procedures, there is a limited body of research on the potential relationship between PFO and adverse outcomes in the context of TLE. This study aimed to evaluate whether the presence of a PFO during TLE is associated with increased rates of stroke, myocardial infarction, or mortality.

## Methods

### Study patients

**Figure 1:**
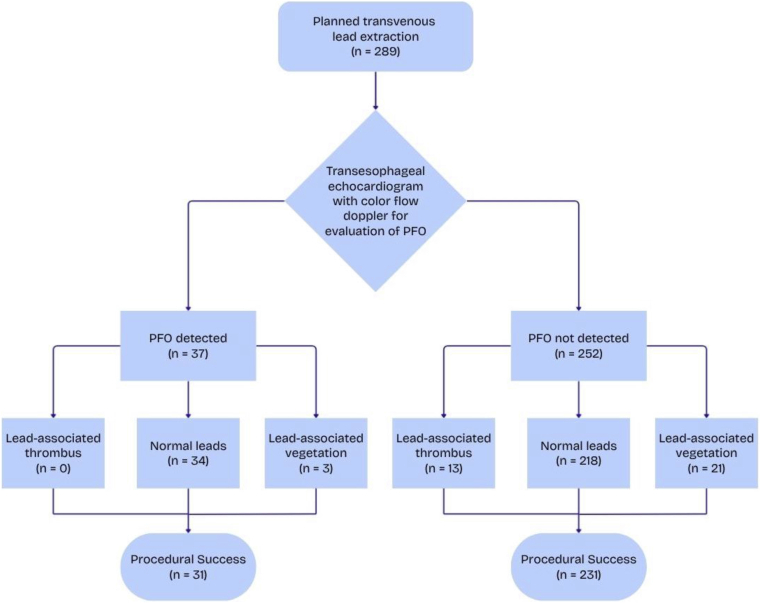
Patient inclusion and categorization flow diagram, with intraoperative transesophageal echocardiogram findings and procedural outcomes. PFO = patent foramen ovale.

We retrospectively evaluated the records of all patients who underwent TLE at the University of California, San Diego, Medical Center between June 2020 and October 2025 to assess outcomes associated with TLEs in patients with CIEDs. Patients were included if they underwent TLE at our institution and had a documented intraoperative transesophageal echocardiogram (TEE) report (Figure 1). The study was approved by the institutional review boards at our hospital and complied with the Declaration of Helsinki as revised in 2013. Our institutional review board approved the use of retrospective data collection without the express consent of each patient given that patient information was deidentified.

### Data collection

**Figure 2:**
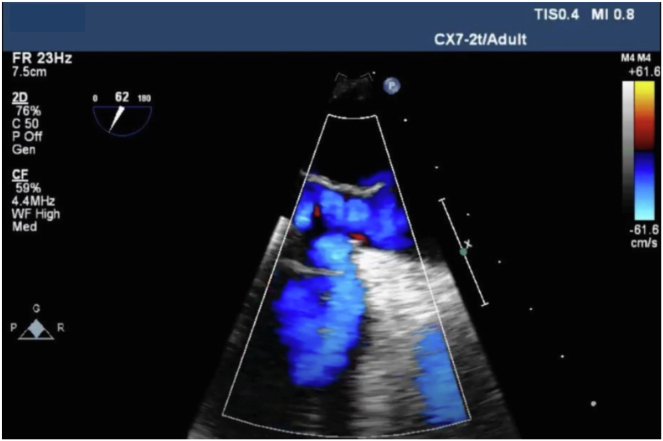
Patent foramen ovale detected during intraoperative transesophageal echocardiogram.

Data were obtained from the electronic health records, including patient demographic, clinical, echocardiographic, and device characteristics, such as device type, number of generator replacements, and lead revisions. Indications for device extraction included infection (pocket and systemic), malfunction (insulation breach or lead fracture), chronic pain, or system upgrade. Procedural data including fluoroscopy time, total procedural time, equipment used, and extraction attempts were reviewed. Intraoperative and postprocedural data also included the presence of a patent foramen ovale (PFO) identified with color flow Doppler during intraoperative TEE (Figure 2), lead-associated thrombus or vegetation, and procedural success. The presence or absence of PFO was assessed immediately before lead extraction and was therefore known to the procedural team before the actual lead extraction; however, the size of the PFO was not reliably commented on. Patients were followed for the duration of their hospitalization during which their TLE procedure was performed, and all in-hospital outcomes were recorded.

### Procedural techniques

The TLE procedure was performed by a cardiothoracic surgeon, with support from an attending electrophysiologist and an anesthesiologist under general anesthesia. TEE was used for monitoring throughout the case, performed under anesthesia in every patient. After appropriate patient preparation, the pectoral device pocket was opened and the pulse generator explanted. A stylet was introduced via the inner lumen. When an active fixation device was used, attempts were made to retract the helix. Manual traction was then applied. If the lead was not freed with gentle traction, the proximal end of the lead was cut, and extraction with a locking stylet (Spectranetics, Colorado Springs, CO, or Cook Medical, Bloomington, IN) was attempted. If extraction by traction was unsuccessful, a laser sheath (Spectranetics) was advanced while short pulses of laser energy were applied to release the lead from endovascular adhesions. If the lead was not freed with the use of a laser, a rotating mechanical sheath (Evolution, Cook Medical, or Tightrail, Spectranetics) was used according to the operator’s discretion.

### Definitions of success and complication

Procedural success was defined according to the Heart Rhythm Society 2017 consensus statement on TLE.^3^ Complete procedural success was defined as “Lead extraction procedure with removal of all targeted leads and all lead material from the vascular space, with the absence of any permanently disabling complication or procedure-related death.” Failure was defined as “Lead extraction procedures in which complete procedural or clinical success cannot be achieved, or the development of any permanently disabling complication, or procedure-related death.” Major complications were defined as those that resulted in death, threatened life, required significant surgical intervention, or caused persistent or significant disability. All other complications attributable to the extraction procedure were considered minor complications.

### Statistical analysis

Statistical analyses were performed using R (version 4.3.3). Descriptive statistics (means and standard deviations for continuous variables; frequencies for categorical variables) were calculated. Fisher’s exact test was used to compare categorical variables between groups, with statistical significance set at *P* < .05. A *t* test was used to compare means of continuous variables between groups, with statistical significance set at *P* < .05. No imputation methods were used for missing data.

In addition, multivariable adjusted logistic regression—controlling for indication for extraction, patient sex, lead dwell time, number of leads extracted, and the presence of thrombus or vegetation on intraoperative TEE—was used to calculate the odds ratios (ORs) and 95% confidence intervals (CIs) for the endpoints of procedural success and stroke. Multivariate regression analysis could not be performed for mortality because there were no deaths in the non-PFO cohort.

A formal sample size calculation was not performed. Instead, all available data from patients who underwent TLE at our institution between June 2020 and October 2025 were included, resulting in 283 patients who underwent 289 procedures being included in the final analysis.

## Results

### Patient characteristics

Overall, 283 patients were included (Table 1). A total of 289 procedures were included, with 37 procedures performed on the PFO cohort and 252 procedures performed in the non-PFO cohort. The cohorts consisted of 283 patients in total, 6 of whom each underwent 2 lead extraction procedures. No significant differences in sex, age, history of heart failure, device type, leads, or indications for lead extraction were noted between cohorts. Most patients underwent lead extraction for a lead malfunction (45.67%) or infection (30.45%). The average lead dwell time was 4.91 years. Although no significant differences were found between cohorts with respect to lead-associated thrombi or vegetations on TEE, notably, no thrombi were found in the PFO cohort on TEE. 3 patients with PFOs were found to have lead-associated vegetations.

**Table 1:** Baseline characteristics, intraoperative characteristics and TEE findings, and postoperative outcomes and complications

Characteristic	Overall (N = 289)	PFO cohort (n = 37)	Non-PFO cohort (n = 252)	*P* value (PFO vs no PFO)	Odds ratio (95% CI), *P* value
Patient age, y, mean ± SD	64.98 ± 15.17	64.59 ± 17.79	65.04 ± 14.79	.8864 (*t* test)	
Female, n (%)	99 (34.26)	12 (32.43)	87 (34.52)	.8549 (Fisher’s)	
Male, n (%)	190 (65.74)	25 (67.57)	165 (65.48)	.8549 (Fisher’s)	
History of CHF, n (%)	163 (56.40)	19 (51.35)	144 (57.14)	.5950 (Fisher’s)	
Explanted device type					
CRT-defibrillator, n (%)	34 (11.76)	2 (5.41)	32 (12.70)	.2774 (Fisher’s)	
CRT-pacemaker, n (%)	12 (4.15)	2 (5.41)	10 (3.97)	.6561 (Fisher’s)	
Defibrillator, n (%)	81 (28.03)	11 (29.73)	70 (27.78)	.8452 (Fisher’s)	
Pacemaker, n (%)	162 (56.06)	22 (59.46)	140 (55.56)	.7245 (Fisher’s)	
Total leads before extraction, mean ± SD	2.14 ± 0.51	2.14 ± 0.42	2.14 ± 0.52	.9610 (*t* test)	
Indication for extraction					
Chronic pain, n (%)	11 (3.81)	1 (2.70)	10 (3.97)	1.0000 (Fisher’s)	
Infection, n (%)	88 (30.45)	14 (37.84)	74 (29.37)	.3393 (Fisher’s)	
Lead/device malfunction, n (%)	132 (45.67)	18 (48.65)	114 (45.24)	.7264 (Fisher’s)	
Need for system upgrade, n (%)	46 (15.92)	4 (10.81)	42 (16.67)	.4741 (Fisher’s)	
TEER, n (%)	2 (0.69)	0 (0.00)	2 (0.79)	1.0000 (Fisher’s)	
Other, n (%)	10 (3.46)	0 (0.00)	10 (3.97)	.3711 (Fisher’s)	
Lead-associated thrombus on intraoperative TEE, n (%)	13 (4.50)	0 (0.00)	13 (5.16)	.3854 (Fisher’s)	
Lead-associated vegetation on intraoperative TEE, n (%)	24 (8.30)	3 (8.11)	21 (8.33)	1.0000 (Fisher’s)	
Lead dwell time, y, mean ± SD	4.91 ± 4.25	5.82 ± 6.22	4.77 ± 3.88	.3258 (*t* test)	
Lead extraction fluoroscopy time, min, mean ± SD	11.25 ± 10.23	13.99 ± 15.92	10.86 ± 9.13	.2636 (*t* test)	
Number of leads extracted, mean ± SD	1.73 ± 0.80	1.81 ± 0.84	1.72 ± 0.79	.5510 (*t* test)	
Procedural success, n (%)	262 (90.66)	31 (83.78)	231 (91.67)	.1324 (Fisher’s)	0.43 (0.16–1.19); *P* = .106
MI, n (%)	0 (0.00)	0 (0.00)	0 (0.00)	1.0000 (Fisher’s)	Unable to calculate
Stroke, n (%)	2 (0.69)	1 (2.70)	1 (0.40)	.2401 (Fisher’s)	3.81 (0.18–79.38); *P* = .388
Death, n (%)	2 (0.69)	2 (5.41)	0 (0.00)	.0160 (Fisher’s)	Unable to calculate
Length of Stay (d), mean ± SD	5.20 ± 9.15	4.30 ± 5.59	5.33 ± 9.56	.3491 (*t* test)	

CHF = congestive heart failure; CI = confidence interval; CRT = cardiac resynchronization therapy; MI = myocardial infarction; PFO = patent foramen ovale; SD, standard deviation; TEE = transesophageal echocardiogram; TEER = transcatheter edge-to-edge repair.

No adjustments were made for confounding variables when calculating Fisher’s exact test and *t* test. When using multivariable regression, we controlled for indication for extraction, patient sex, lead dwell time, number of leads extracted, and the presence of thrombus or vegetation on intraoperative TEE.

### Procedural outcomes and complications

Complete procedural success was achieved in 262 of 289 patients (90.66%). Procedural success rates were similar between the 2 cohorts: 231 of 252 patients (91.67%) in the non-PFO cohort and 31 of 37 patients (83.78%) in the PFO cohort (*P* = .1324). This remained to be the case after multivariate regression (OR 0.43; 95% CI 0.16–1.19; *P* = .106) (Table 2). Complications included the following:

• Stroke: 1 patient in the PFO cohort (2.70%) experienced a stroke compared with 1 patient in the non-PFO cohort (0.40%). Although the rate of stroke was higher in the PFO cohort, the difference between the 2 groups was not statistically significant (Fisher’s exact test *P* = .2401). This remained to be the case after multivariate regression (OR 3.81; 95% CI 0.18–79.38; *P* = .388) (Table 3).
• All-cause mortality: 2 patients in the PFO cohort (5.41%) died during hospitalization, whereas no deaths occurred in the non-PFO cohort (0%). This represents a notable difference in mortality rates between the 2 cohorts. The difference was statistically significant (Fisher’s exact test *P* = .016). Multivariate regression could not be performed because there were no deaths in the non-PFO cohort.

**Table 2:** Multivariate regression analysis for complete procedural success, adjusting for the presence of PFO, patient sex, lead dwell time, total leads, thrombus or vegetation on intraoperative TEE, and indication for extraction

Dependent variable: procedural success			
Independent variable	Odds ratio	95% confidence interval	*P* value
Presence of PFO	0.43	0.16–1.19	.106
Sex (male)	0.83	0.34–2.00	.671
Lead dwell time (y)	1.05	0.94–1.17	.414
Total number of leads	0.8	0.37–1.73	.57
Thrombus on intraoperative TEE	0.47	0.09–2.43	.367
Vegetation on intraoperative TEE	0.65	0.16–2.68	.552
Indication for extraction			
Chronic pain	1	(Reference)	—
Device/lead malfunction	1.18	0.13–10.98	.882
Infection	1.26	0.12–12.65	.846
System upgrade	2.33	0.18–30.73	.521
Other indications∗	0.97	0.05–18.81	.985

Values are presented as odds ratios with 95% confidence intervals. The model included 274 observations. Chronic pain was used as the reference group for the indication for extraction. Model fit was assessed via log likelihood (−85.42) and pseudo R^2^ (0.0314). Combined indications and TEER were omitted from the model owing to a lack of variance/events in those specific subgroups.

PFO = patent foramen ovale; TEE, transesophageal echocardiogram; TEER = transcatheter edge-to-edge repair.

∗ “Other indications” include categories with small sample sizes.

**Table 3:** Multivariate regression analysis for stroke, adjusting for the presence of PFO, patient sex, lead dwell time, total leads, and indication for extraction

Dependent variable: stroke			
Independent variable	Odds ratio	95% confidence interval	*P* value
Presence of PFO	3.81	0.18–79.38	.388
Sex (male)	0.46	0.02–9.67	.62
Lead dwell time (y)	1	0.92–1.08	.937
Total number of leads	0.31	0.02–5.16	.413
Indication for extraction			
Chronic pain	1	(Reference)	—
Infection/malfunction/other∗	1	(Omitted)	—

Values are presented as odds ratios with 95% confidence intervals. The model included 63 observations. Chronic pain was used as the reference group for the indication for extraction. Model fit was assessed via log likelihood (−85.42) and pseudo R^2^ (0.1124). The presence of thrombus or vegetation on intraoperative TEE and subgroups for “Indication for extraction” (including infection, device/lead malfunction, system upgrade, and TEER) were omitted from the final regression calculation because no stroke events occurred within those specific categories, resulting in an undefined odds ratio.

PFO = patent foramen ovale; TEE, transesophageal echocardiogram; TEER = transcatheter edge-to-edge repair.

∗ Include categories with small sample sizes.

Of those who died, the first patient presented in septic shock owing to methicillin-sensitive *Staphylococcus aureus* bacteremia and CIED infection. Intraoperative TEE demonstrated a globular echodense mass on the right atrial lead consistent with vegetation. Despite lead extraction, appropriate antibiotic therapy, and multiple surgical washouts for concomitant right shoulder septic arthritis, the patient remained persistently bacteremic and in refractory shock over the ensuing 2 weeks. After further hemodynamic deterioration after a third operative washout, care was transitioned to comfort measures, and the patient subsequently died.

The second patient was transferred for management of a dehisced pacemaker pocket with lead infection requiring extraction. No pathogen was identified. The procedure was complicated by severe tricuspid regurgitation with shunting through a PFO, which was closed intraoperatively. In the postanesthesia care unit, the patient was found to have right hemiplegia and hemineglect; imaging confirmed a left middle cerebral artery large-vessel occlusion, and emergent thrombectomy was performed. Despite successful revascularization, the patient developed ventricular tachycardia requiring antiarrhythmic therapy and ultimately experienced progressive hemodynamic instability necessitating mechanical ventilation and vasopressor support. After discussions with the family, care was transitioned to comfort measures, and the patient subsequently died.

In the non-PFO cohort, 1 patient had a new stroke identified 26 days after their TLE procedure. The patient had undergone TLE for persistent methicillin-resistant *Staphylococcus aureus* bacteremia and infected leads. 26 days later, they developed altered mental status, and during workup, a head computed tomography demonstrated a new 10 mm lesion in the left uncus. More thorough evaluation with magnetic resonance imaging was unavailable given the presence of a new leadless pacemaker, and given the interval since the procedure, it remained unclear whether this represented a subacute septic embolus, a thromboembolic event, or a primary thrombotic stroke.

## Discussion

This retrospective cohort study aimed to investigate the prevalence of PFO in patients undergoing TLE and compare the rates of adverse outcomes (myocardial infarction, stroke, and mortality) between patients with and without PFO. Our findings suggest that although the rates of myocardial infarction and stroke did not differ significantly between the 2 groups, the mortality rate was notably higher in the PFO cohort (5.41%) than in the non-PFO cohort (0%). This difference in mortality was statistically significant (*P* = .016). However, the inability to calculate an OR and the small sample size in the PFO cohort warrant cautious interpretation.

The higher mortality rate in the PFO cohort may reflect underlying differences in patient characteristics or procedural risks. Although there were no statistically significant differences between the 2 cohorts at baseline, the low event rate limited the ability to use multivariable regression analysis. Notably, previous research has identified advanced age, systemic infection, and reduced left ventricular function as independent predictors of mortality after TLE. For example, Tan et al^2^ highlighted systemic infection as a key risk factor for procedural complications and mortality, whereas Narducci et al^4^ reported a 30-day mortality rate of 7%, with sepsis being the leading cause of death. These findings resonate with our own observation of increased in-hospital mortality in the PFO cohort, 1 of whom died from sepsis after a TLE with a lead-associated vegetation.

Although the precise mechanism linking PFO to increased mortality remains unclear, these results suggest that comorbid conditions and procedural context, including infection, may be major contributors. In the context of our study, the absence of thrombi in the PFO group on TEE and the lack of statistically significant differences in stroke rates between cohorts suggest that embolic phenomena may not be the primary driver of poor outcomes. Instead, these may be manifestations of a more vulnerable clinical profile in the PFO group, potentially exacerbated by sepsis, impaired hemodynamics, or subclinical cardiopulmonary dysfunction. Given the limited sample size and retrospective design, these findings should be interpreted with caution, given that the observed mortality difference may reflect unmeasured confounders rather than a direct causal relationship between PFO and adverse outcomes.

In addition, Narducci et al^4^ emphasized that long-term mortality after TLE was more strongly associated with nonprocedural factors such as renal dysfunction and heart failure than with the technical aspects of the extraction itself. This highlights the possibility that the increased mortality observed in our PFO group could reflect a predisposition to adverse outcomes owing to unmeasured or undetected comorbidities, rather than the presence of PFO alone.

Notably, the lower complete procedural success rate observed in the PFO cohort (83.78%) than the non-PFO cohort (91.67%) and with published benchmarks (≥95% in contemporary series, including Akhtar et al^3^) may reflect several interacting factors. Although individual baseline variables such as lead dwell time and number of leads extracted were not statistically different between groups, both were numerically higher in the PFO cohort, and their combined effect could have contributed to increased procedural complexity. Longer dwell times are known to be associated with greater fibrotic adherence, venous occlusion, and extraction difficulty, whereas multiple leads increase procedural duration and sheath manipulation requirements. These subtle differences, even if not statistically significant in this sample, may collectively explain the lower success rate observed. In addition, institutional and operator-specific factors, including case selection and procedural strategy, may have influenced outcomes.

To the best of our knowledge, our study is the first to evaluate mortality rates specifically in patients with PFO undergoing TLE. Previous research has suggested a link between PFO and complications, specifically stroke, during TLE. For instance, Lee et al^5^ noted that PFO may increase the risk of embolic events during invasive procedures, including TLE. Although our findings align with the theoretical risk, stroke rates did not differ significantly between patients with and without PFO in our cohort. This may be attributable to differences in study design, procedural techniques, or patient selection. Previous literature has also identified TLE as 1 of the highest risk electrophysiological procedures in terms of procedural mortality and cerebrovascular events, defined as both stroke and transient ischemic attack. In a large cohort analysis of nearly 49,000 patients undergoing heart rhythm disorder management procedures, TLE carried the highest mortality rate (1.9%) and the highest incidence of cerebrovascular events (0.21%), with a subset of strokes attributed to suspected paradoxical embolism in patients with PFO.^6^ This finding reinforces our concern that the presence of PFO may confer vulnerability to embolic complications, even if clinically overt strokes were not more prevalent in our cohort.

Furthermore, data from a multicenter registry evaluating more than 11,000 lead extraction procedures reported a perioperative mortality rate of 0.9% and emphasized the importance of advanced procedural planning and cardiothoracic surgical backup.^7^ Although our single-center study achieved a procedural success rate of 90.66%, the mortality rate in patients with PFO (5.41%) far exceeded those reported in broader cohorts, raising the possibility that this subgroup may have unique risks not fully accounted for in generalized TLE risk models.

In other settings, PFO has been associated with worse outcomes during cardiac surgery. Laghlam et al^8^ reported increased ischemic stroke, worsened respiratory outcomes, and longer hospital stays in patients with perioperative PFO. Similarly, Krasuski et al^9^ found no significant differences in mortality or stroke in patients with intraoperative PFO but reported a paradoxically higher stroke rate among those who underwent PFO closure. Such divergent findings may stem from variation in patient characteristics, PFO detection methods, and operative strategies. Our use of intraoperative TEE provides a more accurate method for detecting PFO, adding strength to our findings.

These previous studies emphasize the need for further research to clarify the role of PFO in procedural risks and outcomes. The variability in results underscores the importance of larger, multicenter prospective trials that can better control for confounders and more accurately delineate the impact of PFO on morbidity and mortality in the setting of TLE.

Our study highlights a significant association between PFO and higher in-hospital mortality after TLE, despite similar rates of stroke between groups. This finding suggests that mortality in the PFO cohort may not be primarily driven by embolic events, but instead by other factors—such as hemodynamic compromise, increased vulnerability to sepsis, or the presence of undetected comorbidities. The markedly higher in-hospital mortality rate (5.41% vs 0%) emphasizes the importance of careful preoperative assessment and vigilant postoperative monitoring in patients with PFO undergoing TLE. Clinicians should be especially attentive to warning signs of infection, respiratory dysfunction, and other complications, particularly in patients with predisposing comorbidities such as heart failure or pulmonary disease.

Although stroke was not more common in the PFO cohort, we cannot rule out the contribution of subclinical embolic events, microembolisms, or subtle hemodynamic alterations that could increase risk. Future investigations should explore these possible mechanisms and consider evaluating other markers of procedural outcomes, such as postoperative respiratory metrics or infection severity, which were not captured in this study.

From a clinical perspective, the findings of this study have implications for preprocedural planning and patient counseling. When a PFO is known or detected before TLE, it should be discussed with patients as a potential factor conferring slightly increased procedural risk, particularly regarding embolic or hemodynamic complications. In our institutional practice, perioperative TEE is routinely performed with careful attention to the presence and size of a PFO. If a large PFO is identified, multidisciplinary discussion regarding the potential role of percutaneous closure before extraction may be warranted. Incorporating this information into the informed consent process ensures that patients are aware of potential additional risks and allows for appropriate procedural precautions.

In summary, our findings suggest that PFO may be an important risk factor for mortality during TLE, but the mechanisms behind this association remain unclear. Future studies should investigate whether PFO independently increases the risk of complications or whether the observed outcomes are more closely linked to the comorbidities commonly found in patients with PFO. Understanding this relationship could significantly affect preoperative screening, procedural planning, and postoperative care for this patient population.

### Study limitations

The small sample size in the PFO cohort (n = 37) limits the power of our statistical analyses, particularly for rare outcomes such as stroke and mortality. Consequently, we cannot confidently generalize these findings to larger populations or establish a causal relationship between PFO and increased mortality. The observed prevalence of PFO in our cohort (12.8%) is lower than the approximately 25% prevalence typically reported in the general population. This discrepancy likely reflects the diagnostic modality used; although agitated saline contrast with provocative maneuvers remains the gold standard for PFO detection, our study relied on intraoperative TEE with color flow Doppler. Under general anesthesia and without routine Valsalva maneuvers, smaller or “latent” PFOs may not be visualized. Consequently, our findings likely represent the prevalence of PFOs that are patent under resting procedural conditions, which may be the most clinically relevant in the context of paradoxical embolism during lead manipulation.

Second, missing data on comorbidities such as anticoagulation therapy and the presence of other indwelling hardware may have introduced bias into our results. Third, the retrospective nature of this cohort study inherently limits our ability to establish causality and increases the risk of selection bias. Finally, the low event rate of mortality limited our ability to use multivariable regression analysis to assess for statistically significant differences in mortality, 1 of the primary outcomes of our study.

## Conclusions

Our findings suggest that PFO may be an important risk factor for mortality during TLE but future studies with larger patient populations are needed to further investigate the relationship between PFO and adverse outcomes during TLE. A prospective cohort study could provide more robust data on stroke and mortality incidence in patients with PFO undergoing this procedure. In addition, research into the mechanisms behind the higher mortality observed in patients with PFO—such as the role of hemodynamic changes, arrhythmias, or concurrent comorbidities—would offer valuable insights. Moreover, future studies should assess the impact of PFO closure, the use of cerebral protection devices, or anticoagulation therapy during TLE to evaluate strategies for mitigating these risks.

## Declaration of generative AI and AI-assisted technologies in the writing process

During the preparation of this work, the lead author used OpenAI’s ChatGPT, version GPT-4o, to assist with writing to improve readability and create a more succinct, articulate paper. After using this tool/service, the author reviewed and edited the content as needed and takes full responsibility for the content of the publication.

## Disclosures

Dr Ho reports grant AHA 19CDA34760021 from the American Heart Association, grant NIH 1KL2TR001444 from the National Institutes of Health, Muggleton Family via the Artificial Intelligence Arrhythmia Research Fund at University of California, San Diego, Health, founder shares from Vektor Inc, and being a consultant for Kestra Inc and Medtronic. Dr Birgersdotter-Green received honoraria from Medtronic, Abbott, Biotronik, Boston Scientific, and Philips. Dr Pollema is an educator and proctor for Philips Spectranetics Co. The other authors have no conflicts of interest to disclose.
